# Fatigue in Dental Ceramics: A Call for Standardized Testing and Emerging Manufacturing Technologies

**DOI:** 10.3390/ma19040655

**Published:** 2026-02-09

**Authors:** Gabriel Kalil Rocha Pereira, Rafaela Oliveira Pilecco, João Paulo Mendes Tribst

**Affiliations:** 1 Department of Restorative Dentistry, Faculty of Dentistry, Federal University of Santa Maria (UFSM), Santa Maria 97105-900, RS, Brazil; gabriel.pereira@ufsm.br; 2 Department of Conservative Dentistry, Faculty of Dentistry, Federal University of Rio Grande do Sul (UFRGS), Porto Alegre 90010-150, RS, Brazil; rafaela-pilecco@hotmail.com; 3 Department of Reconstructive Oral Care, Academic Centre for Dentistry Amsterdam (ACTA), Universiteit van Amsterdam and Vrije Universiteit, 1081LA Amsterdam, The Netherlands

In recent decades, dental ceramics have undergone substantial technological and scientific advancement, consolidating their role as key materials for esthetic and functional oral rehabilitation. Improvements in ceramic compositions, processing methods, and digital manufacturing workflows have increased their clinical indications [1]. Despite these advances, the intrinsic brittleness of ceramics and their susceptibility to subcritical crack growth under repetitive loading remain major concerns. Consequently, fatigue behavior is defined as a decisive factor in predicting the long-term clinical performance of ceramic restorations [2].

Recent developments in the field have increasingly emphasized fatigue testing as a more clinically relevant alternative to monotonic load-to-failure tests [3]. Contemporary fatigue protocols aim to simulate oral conditions by incorporating cyclic loading, aqueous environments, and controlled stress amplitudes, enabling a more realistic evaluation of crack initiation, propagation, and failure mechanisms [2,4]. These approaches have strengthened the understanding that fatigue, rather than static strength, plays a significant role in clinical ceramic failures.

However, significant methodological heterogeneity persists. Variations in specimen geometry, loading mode, frequency, number of cycles, environmental conditions, and failure criteria remain widespread in the literature. A previous study highlighted that such heterogeneity limits comparisons among studies and reduces the definition of predictive fatigue models for dental ceramics [2]. Similarly, a scoping review [5] demonstrated that cyclic fatigue tests using non-anatomic specimens, although valuable for mechanistic insights, frequently lack standardization and show limited correlation with clinical behavior. Although non-anatomic specimens allow controlled investigation of material behavior, their predictive value for restoration-level performance is limited [6]. There is a clear need for multilevel approaches that integrate simplified fatigue tests with anatomically relevant models and complementary numerical analyses.

Despite the progress achieved, critical gaps in knowledge are evident. One major gap of concern is the lack of consensus regarding fatigue testing protocols that balance experimental reproducibility with clinical relevance. Although conceptual guidelines and recommendations for fatigue testing exist [4], their adoption across dental materials research is inconsistent. Without proper translation, the laboratory data cannot be used for clinical decision-making.

Parallel to these methodological discussions, another important gap lies in the limited understanding of how different manufacturing routes influence fatigue behavior [7]. While subtractive CAD/CAM ceramics have been extensively studied, new materials are still being released, such as high-translucency zirconia and additively manufactured ceramics, lacking comprehensive fatigue characterization [8]. The long-term implications of processing-induced defects and microstructural anisotropy for slow crack growth and cyclic degradation remain largely unresolved [9].

The emergence of additive manufacturing (AM) technologies has introduced new concepts in ceramic processing. A systematic review [8] demonstrated that AM enables complex geometries, improved material efficiency, and seamless integration into digital workflows. However, the review also emphasized that the mechanical reliability and fatigue behavior of additively manufactured dental ceramics remains insufficiently explored. Layer-by-layer fabrication, residual porosity, anisotropy, and microstructural heterogeneity may significantly affect crack behavior under cyclic loading in the long term, yet robust fatigue data remain scarce.

This Special Issue, “Advanced Dental Materials for Oral Rehabilitation”, was designed to address these challenges by compiling contributions that contribute to the fundamental fatigue principles, methodological considerations, and emerging manufacturing technologies. The eight articles comprising this Special Issue collectively depict the ongoing transition in oral rehabilitation toward materials and strategies that integrate mechanical performance, biological interaction, and clinical functionality.

Taken together, these contributions highlight the trend in dental material research, in which durability, biointegration, safety, and sustainability must be considered together to advance predictable and patient-centered oral rehabilitation [10].

These studies emphasize that fatigue testing is essential for evaluating ceramics. They also highlight current problems with testing methods and suggest ways to improve consistency. By comparing traditional ceramics with new materials and manufacturing methods, this Special Issue shows both progress and challenges. We should look beyond simple mechanical numbers to a more complete understanding that includes material traits, how the ceramic was made, how it is tested, and how it breaks. This approach fits with modern thinking that fatigue is a complex process affected by many factors, not just one parameter [2].

Looking ahead, several key research priorities stand out: first, it is important to better align fatigue testing methods [11]. While full standardization might not be possible, creating agreed-upon guidelines would improve consistency and make results easier to compare. Second, the fatigue behavior of 3D-printed ceramics needs thorough study, especially focusing on anisotropy, defect populations, and long-term use under clinically relevant conditions [8,12]. Third, future research should combine fatigue tests with detailed fracture analysis and computer modeling. This mixed approach can help identify where cracks start, how stresses are distributed, and why failures happen, linking lab results to real-world performance. Finally, turning fatigue data into practical clinical guidelines is a major challenge, requiring studies that connect test results to restoration design, laboratory parameters, pre-processing, cementation strategies, and real service conditions. In summary, continued progress is crucial to ensure that ceramic restorations last long and support evidence-based dental care innovations.
